# The essential habitat role of a unique coastal inlet for a widely distributed apex predator

**DOI:** 10.1098/rsos.230667

**Published:** 2023-10-11

**Authors:** Agustín M. De Wysiecki, Adam Barnett, Federico Cortés, Rodrigo Wiff, Pablo J. Merlo, Andrés J. Jaureguizar, Cynthia A. Awruch, Gastón A. Trobbiani, Alejo J. Irigoyen

**Affiliations:** ^1^ Centro para el Estudio de Sistemas Marinos, Consejo Nacional de Investigaciones Científicas y Técnicas (CONICET), Puerto Madryn, Chubut, Argentina; ^2^ Marine Data Technology Hub, James Cook University, Townsville, Queensland, Australia; ^3^ Biopixel Oceans Foundation, Cairns, Queensland, Australia; ^4^ Instituto Nacional de Investigación y Desarrollo Pesquero, Mar del Plata, Buenos Aires, Argentina; ^5^ Center of Applied Ecology and Sustainability (CAPES), Pontificia Universidad Católica de Chile, Santiago, Chile; ^6^ Instituto Milenio en Socio-Ecología Costera (SECOS), Santiago, Chile; ^7^ Comisión de Investigaciones Científicas de la Provincia de Buenos Aires (CIC), La Plata, Buenos Aires, Argentina; ^8^ Instituto Argentino de Oceanografía (IADO), Bahía Blanca, Buenos Aires, Argentina; ^9^ Universidad Provincial del Sudoeste (UPSO), Coronel Pringles, Buenos Aires, Argentina; ^10^ Fisheries and Aquaculture, Institute for Marine and Antarctic Studies (IMAS), University of Tasmania, Hobart, Tasmania, Australia

**Keywords:** acoustic tracking, aggregation, marine protected area, sevengill shark, spatial ecology, tidal effect

## Abstract

Essential habitats support specific functions for species, such as reproduction, feeding or refuge. For highly mobile aquatic species, identifying essential habitats within the wider distribution range is central to understanding species ecology, and underpinning effective management plans. This study examined the movement and space use patterns of sevengill sharks (*Notorynchus cepedianus*) in Caleta Valdés (CV), a unique coastal habitat in northern Patagonia, Argentina. Seasonal residency patterns of sharks were evident, with higher detectability in late spring and early summer and lower during autumn and winter. The overlap between the residency patterns of sharks and their prey, elephant seals, suggests that CV functions as a seasonal feeding aggregation site for *N. cepedianus*. The study also found sexual differences in movement behaviour, with males performing abrupt departures from CV and showing increased roaming with the presence of more sharks, and maximum detection probability at high tide. These movements could be related to different feeding strategies between sexes or mate-searching behaviour, suggesting that CV may also be essential for reproduction. Overall, this study highlights the importance of coastal sites as essential habitats for *N. cepedianus* and deepens our understanding of the ecological role of this apex predator in marine ecosystems.

## 1. Introduction

Mobile apex predators can have significant effects on ecosystem dynamics [1]. Cross-system movements by mobile predators can connect multiple habitats and regions [2,3], transporting energy and biomass across extended areas and affecting nutrient cycling and productivity [4,5]. Long-distance dispersal can also facilitate gene flow between distant places [6]. When present in a particular area, predators influence communities by consuming prey and inducing risk effects on both prey and competitors [7,8]. This, in turn, affects the local diversity and food-web dynamics, contributing to ecosystem architecture and functioning [9,10]. The disproportionately important roles that apex predators play in the ecosystem stability and functioning suggest they are a central component of ecosystem health and resilience [11].

While the ecological roles of highly mobile predatory sharks are particularly pronounced in areas where they aggregate, these areas represent ‘essential habitats' for their survival. Essential habitats are areas that support specific functions (e.g. reproduction, feeding, refuge) over various life-history stages of a species [12]. Therefore, knowledge of the contribution of habitats to the survival and success of various life-history stages is fundamental to understanding ecosystem function and dynamics, and contributing to species and ecosystem management. Temporal uses of coastal aggregation areas (e.g. bays, mangroves, coastal reefs) are common among shark species and the drivers of these temporal coastal aggregations vary. Some species aggregate in large numbers in foraging grounds, targeting seasonally abundant prey [13,14]. Other species' aggregation behaviour is linked to reproduction [15]. Juvenile individuals aggregate in shallow bays and estuaries that may provide safe habitat from larger predators [16,17]. Inshore aggregations have also been associated with behavioural thermoregulation to increase metabolic rates [18,19]. Sharks commonly display site fidelity to such aggregation areas, often returning annually to fulfil ecological requirements [20]. Annual aggregations in discrete habitats characterizes the life history of many shark species and is central to the maintenance of their populations over time.

The higher density of individuals at aggregation sites provides unique opportunities to deepen our knowledge of the ecology of many highly mobile shark species, that otherwise would be difficult to study. For example, studies incorporating spatial use at aggregation sites have contributed to estimating demographic parameters such as abundance and survival rates [21,22], revealing fine-scale movement behaviour [23,24], unravelling complex predator–prey relationships [13,25] and generally increasing knowledge on habitat use patterns [26]. Such information has been applied to the design and improvement of marine reserves [27,28]. The collection of spatial data integrated with other ecological information is, therefore, improving our ability to preserve key habitats for sharks and their ecological functions [29,30].

The broadnose sevengill shark (*Notorynchus cepedianus*) is a key predator influencing ecosystem dynamics in temperate coastal systems of the world [31–33]. The high trophic level diet of *N. cepedianus* includes teleost, chondrichthyans and marine mammals [34–37]. Annual seasonal movements into discrete coastal habitats, at least in some locations, are driven by *N. cepedianus* returning to productive foraging grounds [33,38–41]. Large-scale (greater than 1000 km) seasonal migratory movements away from and returning to forging grounds [37,42,43] implies these coastal habitats are essential for supporting adult *N. cepedianus*.

In early 2021, an *N. cepedianus* male individual tagged with an external identification tag (spaghetti tag) in Caleta Valdés (CV) was recaptured in Rocas Coloradas, southern Argentina (approx. 490 km away), representing the first direct evidence of large-scale movement of the species in the Southwest Atlantic (A. Irigoyen, personal communication, 2021, electronic supplementary material, figure S1). Maps of potential distribution support a regional core home range and large-scale seasonal displacements of *N. cepedianus* connecting distant areas between southern Brazil and southern Argentina [44,45]. Given the capacity to migrate along the southeast coast of South America, determining when and what drives *N. cepedianus* use of specific habitats is of interest. Parturition events in *N. cepedianus* have been suggested to play a role in driving movements and habitat use [44]. Use of specific coastal habitats may be associated with targeting diverse prey across marine inlets to which *N. cepedianus* display site fidelity [40,46].

*Notorynchus cepedianus* seasonally aggregates in CV, a semi-isolated shallow marine inlet in northern Patagonia (Argentina). The CV's distinctive characteristics include long-stretch gravel bank separation from and narrow opening to the ocean, a strong tidal regime, and a high concentration of marine fauna [47]. The site has been recently recognized as a key aggregation site for *N. cepedianus*, with high abundance between late spring and early summer [40]. During this period, spontaneous regurgitations of stomach contents by individuals captured during fishing surveys confirmed that southern elephant seal (*Mirounga leonina*) is an important component in the sharks’ diet [40,48]. Adult elephant seals also display seasonal variation in abundance with a peak in spring when females arrive to breed and moult [49,50]. Weanling pups stay until December when they dive for the first time [49]. Given the importance of marine mammals to *N. cepedianus* diets globally [36], and previous studies linking the occurrence and abundance of *N. cepedianus* to seasonal habitat use of key prey species [13], elephant seal habitat use patterns could potentially be a strong driver for *N. cepedianus* seasonal abundance in CV [40]. Alternatively, or perhaps simultaneously, CV has also been hypothesized as a mating ground for *N. cepedianus*. This is based on several females with fresh mating scars and reproductive hormone levels associated with the timing of mating in both sexes [40,51].

The overall aim of this study is to investigate adult *N. cepedianus* habitat use of CV. First, we determine the temporal use (seasonal residence) of CV. Second, we examine differences in fine-scale habitat use between sexes within the inlet. Third, we investigate environmental and biological drivers in the use of the area. Finally, we discuss the significance of CV for *N. cepedianus* in the context of other bays and inlets studied within its distribution range in the Southwest Atlantic.

## 2. Material and methods

### 2.1. Study site

**Figure 1.**
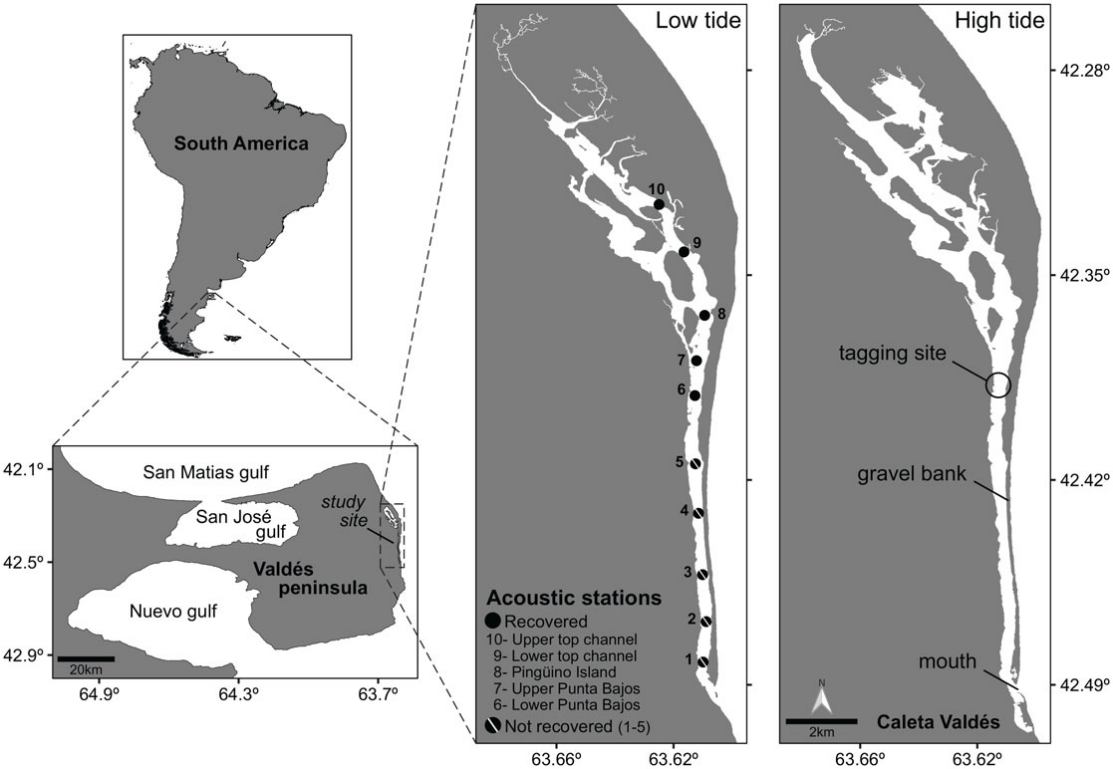
Location of the study site of Caleta Valdés in northern Patagonia, Argentina. Contrasting scenarios of low and high tides in the area are included. The spatial polygons were constructed using Google Earth satellite images (low tide on 18 September 2013 and high tide on 15 October 2018) and Qgis.

The CV is a marine inlet located within the protected area of ​Valdés Peninsula in the Chubut Province (northern Patagonia, Argentina; figure 1). The inlet is approximately 30 km long, has a north–south orientation, and is separated from the ocean by a large gravel bank [52]. A small but dynamic opening of a few hundred metres at the southern end allows marine water to enter and exit the site. The inlet is characterized by its narrow width (range 200–700 m) and shallow depth (down to 13 m) and by strong currents (range 7.5–11 m s^−1^) of changing direction due to the tidal cycle [47]. The upper end of CV is shallower, less energetic, has a greater deposit of sediments, and consists of islets with vegetation that connect and disconnect every day depending on the tides (see low-high tide contrast in figure 1). By contrast, the topography across the lower end of CV creates a bottleneck of deeper waters and stronger currents, leading to a more dynamic habitat with bare bottoms. The water surface can cover an area of 15.71 km^2^ during low tide, increasing to 19.43 km^2^ during high tide (figure 1). The surface water temperature varies seasonally between a minimum value of 8°C in August and a maximum of 18°C in February (Aqua MODIS, 2022, data not published).

### 2.2. Acoustic array

An array of ten omnidirectional acoustic receivers (Vemco VR2W—69 kHz) was arranged almost equidistant (approx. 2 km) along CV on 29 October 2019 (figure 1). Each receiver was fixed to a stainless-steel pipe with a base weighing an average of 70 kg. Each base was joined by a 10 m rope to a second 25 kg square cement structure to reinforce its fixation and facilitate its subsequent recovery. To maximize coverage range, the ten structures were deployed from a boat near the midpoint of the inlet. Given that the CV hardly exceeds 300 m in width along its entire length and that receivers have an average effective coverage range of up to 400 m in all directions [53], it was assumed that each receiver functions as a ‘gate’. Unfortunately, five of the ten moorings did not hold the drag of the water currents and were never located nor were receivers recovered. The lost receivers were initially deployed at the lower half of CV where the tidal energy is higher (figure 1). The remaining five receivers were located and removed twice throughout the study period (i.e. 30 January 2020 and 15 November 2020) for less than 48 h to allow for data downloads and software updates. Due to the loss of five receivers, the analyses were restricted to the upper half of CV.

### 2.3. Tagging procedures

Sevengill sharks were caught using rods and bottom longlines in various locations within a 1000 m radius from Punta Bajos (42°23′16″S, 63°36′51″W; figure 1) on three consecutive days (29–31 October 2019). Bottom longlines consisted of a 70 m lead-core mainline with 0.8 m stainless-steel snoods and five hooks (Mustad 2330-DT, size 1). Atlantic chub mackerel (*Scomber colias*) was used as bait. Longlines were set from a small inflatable boat and then operated from the shore. Soak time was limited to periods between 60 and 90 min or until a shark was felt hooked to the longline. Once on shore, the hook was removed and each individual was measured for total length (*L*_T_), sexed and tagged externally with a conventional tag (model FT-1-94, Floy Tag). Sharks were manoeuvred to a tonic immobility position with their gills underwater. Individuals underwent abdominal surgery to implant the acoustic transmitter (Vemco V16 coded transmitter, 60–180 s emission interval, *ca* 10 years battery life) into the peritoneal cavity through a 2–3 cm incision. The incision was then sutured using a needle and biodegradable surgical thread (absorbable polyglycolic acid, Safil).

### 2.4. Data curation

Before the analyses, the detection dataset was screened for collision detections, as well as single detections per day at any particular receiver, which were removed to avoid potential false detections. To rule out any potential tag loss from unhealed wounds, and both tagging-induced mortality or permanent migration, sharks detected in the array only within the first 5 days in total were considered non-representative of any movement pattern and were excluded from the analyses. The first three tagging days were also not considered in the analyses to avoid the potential effect of post-tagging stress on the initial detections. All data processing and analyses were conducted in R software [54], and the code is available on GitHub (Agustindewy/Sevengill_shark_telemetry).

### 2.5. Seasonal analyses

We examined patterns of residency, movement and space use over a period of 15 months to understand fine-scale habitat use by *N. cepedianus* in CV. Because the CV is a seasonal aggregation area for the species, the study period was first classified into high and low abundance periods (herein ‘high season’ and ‘low season’). Based on rod and reel and camera-derived abundance indices, *N. cepedianus* aggregates in high numbers between mid-spring and mid-summer (i.e. October to February, high season), whereas for the rest of the year, its abundance remains lower (i.e. March to September, low season) [40,55]. To corroborate this, we calculated the monthly distribution of the average proportion of tagged sharks present per day and receiver. To avoid the effect on abundance from sharks permanently leaving CV, we considered the daily proportion of detected/remaining sharks in the array. This proportion represents the number of sharks detected on a particular day relative to the sharks that are either present or absent on that day but still have not permanently left the array.

#### 2.5.1. Residency and roaming indices

We used the daily occurrence of tagged sharks to explore the seasonal residency of the species within the CV. Two indices were considered to account for uncertainty in residency behaviour supported by the detection data [56]. The maximum residency index was calculated by dividing the number of days a tagged shark was detected at the array by the total number of days from its first detection to its last detection. This measure may overestimate residency as it does not consider the possibility of undetected days from its last detection to the end of the study. By contrast, the minimum residency index was calculated by dividing the number of days each tag was detected at the array by the total duration of the study period (i.e. 497 days). This measure may underestimate residency because, after the last detection and before the end of the study period, there exists the possibility that the tag could have been lost due to various factors, such as the shark's death, predation or permanent departure from the area. Therefore, these indices provide strict measures of the minimum and maximum residency behaviour a particular shark could have possibly developed during the study. The residency index ranged from 0 to 1, with values close to 0 indicating low residency and values close to 1 representing high residency.

Movement patterns were described using a roaming index, which indicates the extent an individual moved within the receiver array. This index is calculated on a daily temporal scale as the average proportion of receivers an individual visited during the days it was present in the array [57]. Because the array was composed of five receivers, the roaming index ranged from 0.2 to 1, with a value of 0.2 indicating minimum roaming (one receiver visited) and a value of 1 representing maximum roaming (five receivers visited).

In addition to describing residency and roaming for each shark individual considering the whole monitoring period, we used temporally binned measures of both indices to explore differences between the high and low seasons of shark abundance determined above. The indices were used to categorize shark behaviour either as a vagrant (less than 0.1, low residency), short-term resident (0.1–0.5, moderate residency) or long-term resident (greater than 0.5, high residency), and whether they presented different roaming behaviour in each season [58]. Residency (minimum) and roaming scores were plotted together to compare residency and movement patterns between the different abundance seasons.

#### 2.5.2. Detection probability

A generalized additive mixed model (GAMM) was used to model the probability of shark detection. For each tagged shark, we constructed a binary response variable using daily detection data (1 for detected, 0 for not detected) from the start of the monitoring period to the last detection day. The model included sex, total length (cm), month, sea surface temperature (°C) and tide amplitude (m) as smoothed fixed terms. The individuals identified with a unique tag number (hereafter ‘tag’) were modelled as random components. Daily sea surface temperature at approximately 4 km resolution was obtained from Aqua MODIS (https://coastwatch.pfeg.noaa.gov/) and tide amplitude was calculated as the difference between the low and high tide measures from the nearest tide gauge (Comodoro Rivadavia, http://www.hidro.gov.ar/). To account for potential sexual segregation, we tested a model including an interaction term with month and sex. We also included the tag and its interaction with sex as random effects to account for variation in detection between individual tags and the repeated nature of the data. A cyclic cubic spline was used to model the month and temperature to account for their cyclic seasonal nature. Given the binary nature of the response variable, we implemented a GAMM with a binomial link function using the ‘mgcv’ package in R [59]. We estimated the model parameters with restricted maximum likelihood (REML). The inclusion of each explanatory variable and the best model selection were assessed with the Akaike information criterion using the ‘MuMIn’ package [60].

#### 2.5.3. Roaming behaviour

A roaming index was constructed by counting the number of receivers a tagged shark visited and did not visit during the days it was present in the array between the beginning of the monitoring period and the last detection day. To test the effect of same-sex and opposite-sex shark presence on the roaming index, we constructed daily variables named ‘prop_f’ and ‘prop_m’, which represent the proportion of detected/remaining females and males in the array, respectively. This proportion represents the number of sharks of each sex detected on a particular day relative to the total number of sharks of that sex that have not permanently left the array up to that day. The roaming index was modelled using a GAMM with binomial error distribution. The global model included sex, total length, season, prop_f and prop_m (and interaction of both with sex), sea surface temperature and tide amplitude as smoothed fixed terms. Individuals identified with a unique tag number were modelled as random components.

#### 2.5.4. Space use

Differences in patterns of space use between females and males were analysed using dynamic Brownian bridge movement models (dBBMM) to incorporate temporal and behavioural characteristics of movement paths into the estimation of the home range [61]. Due to the intricate topography of CV, we calculated the dBBMM of each sex group accounting for land contours for more realistic estimations of space use, using the RSP package [62]. We reconstructed the movement tracks by interpolating consecutive detections in different acoustic receivers within an interval of a minimum of 5 min and a maximum of 24 h; detections were broken into a new track for sharks not detected for a longer period than 24 h. We binned these tracks by abundance season and used them to calculate the 50% dBBMM areas (in m^2^), which are the areas where females and males spent half of their tracking times. Finally, the dBBMM areas were overlaid and plotted to assess possible differences in space use between sex and how it was affected by the seasonal change in the abundance of sharks.

### 2.6. Intra-day analyses

A strong tidal action dominates the water current dynamics of the area, and during high tide, the water reaches tidal plains and channels across upper sections [47]. This raised the question of whether the height, direction and/or strength of tide may be important drivers of the intra-day use of CV. Therefore, we were interested in testing how tidal action may affect the use of CV by *N. cepedianus* within the 24 h cycle. To this end, we explored the hourly residency patterns of sharks and evaluated how they are affected by the tide metrics in the area.

#### 2.6.1. Hourly detection

Detections were treated as circular data over a 24 h period to explore possible hourly detection patterns. Rao's homogeneity test was first used to determine whether there was significant variation in the hourly patterns among individuals. Rao's spacing test was then used to determine whether circular data for each individual was uniformly distributed or biased to a particular time of day. Both tests were performed using the ‘circular’ package [63]. In addition, we used hierarchical cluster analysis (‘average’ method) to differentiate individuals given their hourly detection patterns. A heat map was then generated to visually compare the hourly detection patterns among the clusters.

#### 2.6.2. Tidal effect

A GAMM was used to investigate the tidal effect on detection probability at the intra-day scale. For each tagged shark, we constructed a binary response variable using hourly detection data (1 for detected, 0 for not detected) during the days it was present in the array between the beginning of the monitoring period and the last detection day. Since current metres could not be used in this study nor *in situ* tidal measurements exist for the area, we focused on the overall tidal action derived from astronomic predictions. Three categorical variables were constructed using hourly tidal data (metres), including the height, direction and strength of the tide. The tide height was classified as ‘low’, ‘intermediate’ or ‘high’ based on their 0.25 and 0.75 quantiles. The tide direction was classified as ‘inflow’ when tides increased or ‘outflow’ when tides decreased. The tide strength was classified as ‘weak’ in moments when tides changed direction and the difference in tide height was −0.5 > 0 < 0.5, or ‘strong’ otherwise. The model included total length, season, hour of the day (and its interaction with sex) as smoother effects, while the sex and the three tidal metrics (and their interaction with sex) were modelled as fixed categorical terms. Hourly tidal data were obtained from astronomic predictions for the nearest main port (Puerto Madryn, http://www.hidro.gov.ar/). To validate and correct the astronomic predictions, we used three past tidal datasets measured *in situ* in CV (at Punta Bajos) using data loggers (15–16 August 2015, 14–15 November 2015 and 12–13 March 2016). A cyclic cubic spline was used to model the hour of the day to account for its cyclic daily nature. The tag was also included as a random effect.

## 3. Results

Twenty sevengill sharks were fitted with acoustic transmitters and monitored between 1 November 2019 and 12 March 2021 (i.e. 497 days). Between the first monitoring day and the end of the study period, acoustic signals from one tag (female 225 cm total length) became stationary within the range of the acoustic receiver at Isla Pingüino, suggesting tag loss/expulsion. Another female (no. 5973) was detected for only two days within the first five days of the study and was removed from the statistical analyses. The remaining 18 individuals were all adults (11 females, 7 males) ranging in size between 183 and 245 cm total length, were detected for periods of 78–497 days (281.4 ± 181.5 days), and travelled minimum distances between 40.5 and 3093.1 km (913.2 ± 785.3 km) (electronic supplementary material, table S1).

### 3.1. Seasonal patterns

**Figure 2.**
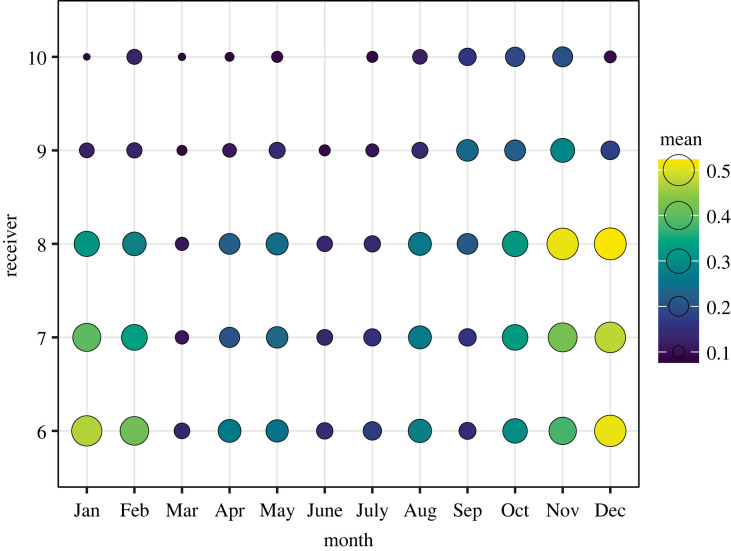
Daily average proportion of tagged sevengill sharks (*Notorynchus cepedianus*) detected at each receiver within the array, binned by month. Location of receivers in figure 1.

The daily average proportion of tagged sharks detected within the array binned by month confirmed the peak of relative abundance between October and February (i.e. high season), with the highest values in December (figure 2). The high season was notable only across the three lower receivers at Pingüino Island, upper Punta Bajos and lower Punta Bajos. Lower proportions of tagged sharks occurred in the rest of the year indicating a lower abundance in the area between March and September (i.e. low season). Based on this result, tagged sharks were monitored for two high seasons (November to February 2019/2020 and October to February 2020/2021) and one low season (March to September 2020).

**Figure 3.**
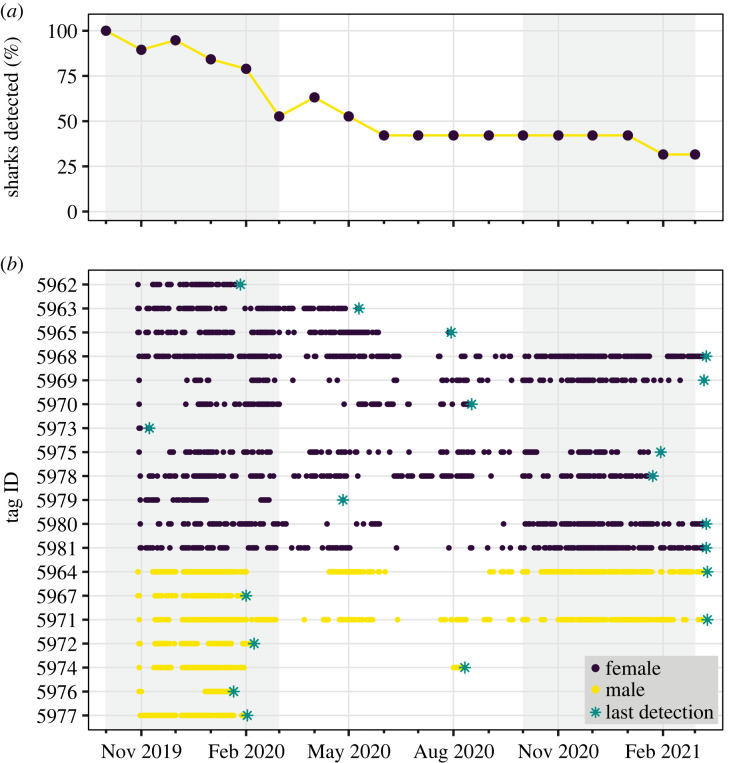
Detection history of tagged sevengill sharks (*Notorynchus cepedianus*) detected at the array expressed as the percentage of sharks detected by month (*a*) and raw detection data (*b*). Shaded areas represent the high abundance seasons.

The percentage of sharks detected each month decreased steadily throughout the study period, reaching values lower than 50% after seven months (figure 3*a*). However, individuals generally resided in CV for long but interrupted periods during the detection window before they permanently left the array (mean ± s.d. minimum residency: 0.211 ± 0.145, mean ± s.d. maximum residency: 0.448 ± 0.219, electronic supplementary material, table S1, figure 3*b*). Each time an individual entered the array, it remained within the range of receivers for at least two consecutive days up to a period of 46 consecutive days (mean ± s.d.: 17.1 ± 9.9, electronic supplementary material, table S1). Conversely, each time an individual temporarily left the array, it remained out of range for a minimum of five consecutive days and a maximum of 183 days before being detected again (mean ± s.d.: 53.4 ± 45.4, electronic supplementary material, table S1). Five sharks (one female, four males) permanently left the array by the end of the first high season (October to February 2019/2020) and only eight (six females, two males) were detected until the end of the next high season (October to February 2020/2021, figure 3*b*). Most males (six out of seven) departed abruptly from CV before the end of the high season. All six males departed in a period of 18 days, four in 73 h and two just under 4 h (figure 3*b*). By contrast, a lower proportion of females (4 out of 12) left the area more gradually by the end of the high season and stayed for longer periods during the low season (March to September 2020) (figure 3*b*). Sharks exhibited moderate to high roaming behaviour (range 0.389–0.700) when considering the full detection window for each individual (electronic supplementary material, table S1).

**Figure 4.**
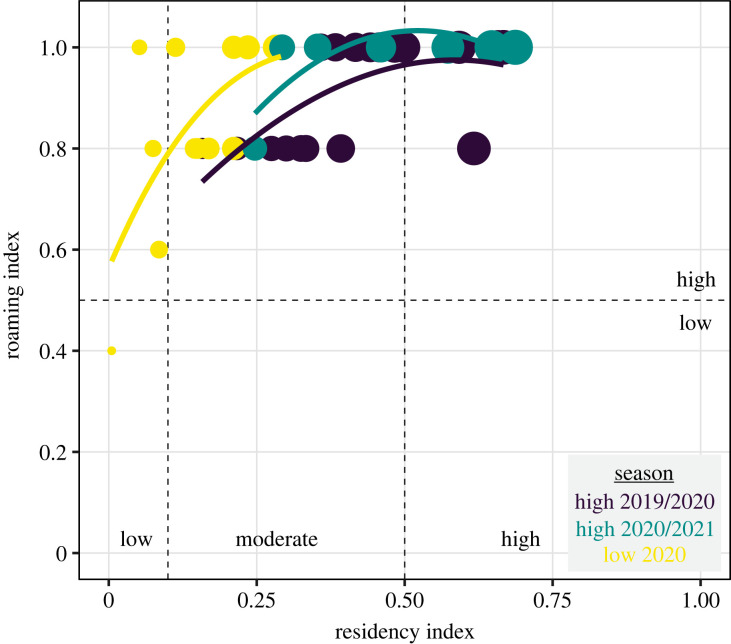
Seasonal variation in the relationship between residency (minimum) status and roaming behaviour for tagged sevengill sharks (*Notorynchus cepedianus*). Dashed lines delineate categories in each variable. Solid colour lines represent loess smoother fits to the points in each season.

Tagged sharks exhibited a moderate to high residency during the high seasons (minimum residency 0.16–0.69), as opposed to the low season when they showed low to moderate values (minimum residency less than 0.3, figure 4). This pattern indicated sharks behaved as long-term and short-term residents during the high season, while they behaved as short-term or vagrant residents during the low season. In general, sharks exhibited a high roaming behaviour (≥0.8) across the study period (figure 4). However, a few sharks visited fewer receivers (three or fewer) during the low season, whereas all sharks detected during the high seasons visited four or five receivers (figure 4).

**Figure 5.**
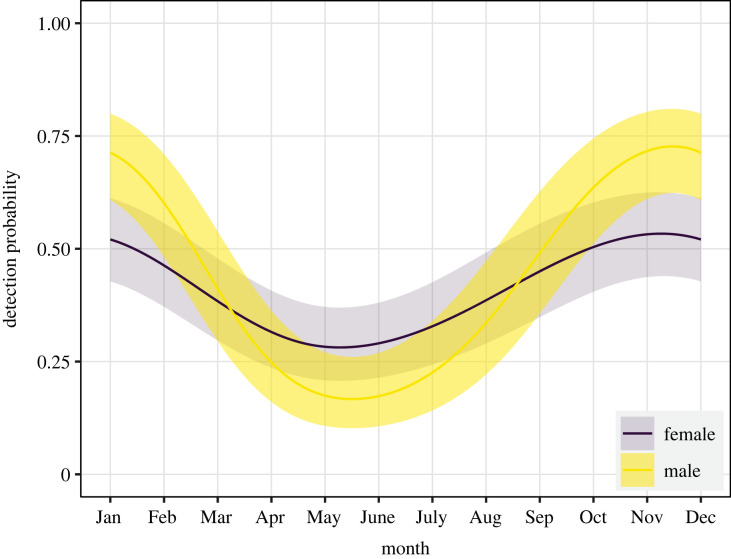
Detection probability of female and male sevengill sharks (*Notorynchus cepedianus*). Solid lines represent the mean prediction and shaded areas in the 95% confidence interval.

Based on the GAMM model selection, the inclusion of the month interaction with sex and the tag as a random effect led to the best model fit for the seasonal detection probability (table 1). The final model included sex, month, month:sex interaction, sea surface temperature, tide amplitude and tag (table 1). All predictors, except female interaction with month, affected shark detection probability, indicating that the seasonal changes were more pronounced in males compared to females (table 2). In general, individuals showed a higher detection probability (greater than 0.5) between October and January, with a maximum between November and December and a minimum between May and June (figure 5). The maximum detection probability of both sexes occurred at 14–15°C surface temperature and higher tide amplitude (electronic supplementary material, figure S2).

**Table 1.** Ranked models showing factors affecting the seasonal and intra-day detection probability and roaming behaviour of sevengill sharks (*Notorynchus cepedianus*) in Caleta Valdés array. Models were ranked by the Akaike information criterion corrected by sample size (AICc). Bold font indicates simplest best-ranked models. d.f., degrees of freedom; Dev., explained deviance (%); TL, total length (cm); SST, sea surface temperature (°C); TA, tide amplitude (m); Prop_f, proportion of detected/remaining females in the array; Prop_m, proportion of detected/remaining males in the array.

fixed factors	random factors	d.f.	AICc	ΔAICc	dev.
seasonal detection probability (*s.detec*)					
*s.detec* ∼ Sex + s(TL) + s(Month) + s(Month:Sex) + s(SST) + s(TA)	Tag:Sex	27.1	6143.8	0.0	14.6
*s.detec* ∼ Sex + s(TL) + s(Month) + s(Month:Sex) + s(SST) + s(TA)	Tag	27.1	6144.2	0.4	14.5
***s.detec* ∼ Sex + s(Month) + s(Month:Sex) + s(SST) + s(TA)**	**Tag**	**27.1**	**6144.2**	**0.4**	**14.5**
*s.detec* ∼ Sex + s(TL) + s(Month) + s(SST) + s(TA)	Tag	25.1	6196.2	52.2	13.8
*s.detec* ∼ Sex + s(TL) + s(Month) + s(Month:Sex) + s(SST) + s(TA)	—	18.7	6272.9	129.1	12.5
seasonal roaming index (*s.roam*)					
*s.roam* ∼ Sex + s(TL) + Season + s(Prop_f) + s(Prop_f:Sex) + s(Prop_m) + s(Prop_m:Sex) + s(SST) + s(TA)	Tag	25.8	5631.1	0.0	16.6
***s.roam*** ∼ **Sex + s(Prop_f) + s(Prop_f:Sex) + s(Prop_m) + s(Prop_m:Sex) + s(SST)**	**Tag**	**25.8**	**5631.3**	**0.2**	**16.6**
*s.roam* ∼ Sex + s(TL) + Season + s(Prop_f) + s(Prop_f:Sex) + s(Prop_m) + s(Prop_m:Sex) + s(SST) + s(TA)	Tag:Sex	26.1	5631.7	0.6	16.6
*s.roam* ∼ Sex + s(TL) + Season + s(Prop_f) + s(Prop_f:Sex) + s(Prop_m) + s(Prop_m:Sex) + s(SST) + s(TA)	—	18.0	5673.7	42.6	14.1
*s.roam* ∼ Sex + s(TL) + Season + s(Prop_f) + s(Prop_m) + s(SST) + s(TA)	Tag	24.1	5709.9	78.8	13.0
Intra-day detection probability (*i.detec*)					
***i.detec*** ∼ **Sex + s(Hour) + s(Hour**:**Sex) + s(Tide_height) + s(Tide_height**:**Sex) + Tide_direction + Tide_direction:Sex**	**Tag**	**36**.**3**	**53579**.**0**	**0**.**0**	**5**.**1**
*i.detec* ∼ Sex + s(TL) + s(Hour) + s(Hour:Sex) + Tide_height + Tide_height:Sex + Tide_direction + Tide_direction:Sex	Tag	36.2	53579.0	0.0	5.1
*i.detec* ∼ Sex + s(TL) + s(Hour) + s(Hour:Sex) + Tide_height + Tide_height:Sex + Tide_direction + Tide_direction:Sex	Tag:Sex	36.2	53579.5	0.5	5.1
*i.detec* ∼ Sex + s(TL) + s(Hour) + s(Hour:Sex) + Tide_height + Tide_height:Sex + Tide_direction + Tide_direction:Sex	—	29.2	53933.8	354.8	4.4
*i.detec* ∼ Sex + s(TL) + s(Hour) + s(Hour:Sex) + Tide_height + Tide_direction	—	25.1	54227.5	648.5	3.9
*i.detec* ∼ Sex + s(TL) + Season + s(Hour) + Tide_height + Tide_direction + Tide_strength	—	22.5	54254.3	675.3	3.8
*i.detec* ∼ Sex + s(TL) + s(Hour) + Tide_height + Tide_direction	—	20.3	54255.0	676.0	3.8

**Table 2.** Best generalized additive mixed models showing seasonal and intra-day effects on detection probability and roaming behaviour of sevengill sharks (*Notorynchus cepedianus*) in Caleta Valdés. Prop_f, proportion of detected/remaining females in the array; Prop_m, proportion of detected/remaining males in the array; edf, estimated degrees of freedom; s, smooth function.

fixed terms	estimate	s.e.	*p*-value	smooth terms	edf	*F*-statistic	*p*-value
*seasonal detection probability*							
intercept	−0.834	0.191	*<0.001*	s(Month)	1.9	170.1	*<0.001*
sex male	0.277	0.312	0.374	s(Month):Sex female	0.0	0.0	0.201
				s(Month):Sex male	1.9	119.3	*<0.001*
				s(Tide amplitude)	2.6	16.9	*<0.001*
				s(Sea surface temperature)	2.0	212.9	*<0.001*
				s(Tag)	15.0	248.1	*<0.001*
*seasonal roaming index*							
intercept	−0.091	0.068	0.182	s(Prop_f)	0.0	0.0	0.653
sex male	0.395	0.124	*0.001*	s(Prop_f):Sex female	0.0	0.0	0.777
				s(Prop_f):Sex male	1.0	40.1	*<0.001*
				s(Prop_m)	0.0	0.0	0.911
				s(Prop_m):Sex female	0.0	0.0	0.912
				s(Prop_m):Sex male	5.6	43.3	*<0.001*
				s(Sea surface temperature)	2.5	75.5	*<0.001*
				s(Tag)	12.8	87.1	*<0.001*
*intra-day detection probability*							
intercept	−1.193	0.095	*<0.001*	s(Hour)	3.4	6.3	*<0.001*
sex male	1.438	0.152	*<0.001*	s(Hour):Sexfemale	3.1	5.3	*<0.001*
Tide_height intermediate	−0.074	0.034	0.03	s(Hour):Sexmale	3.7	10.3	*<0.001*
Tide_height low	−0.149	0.041	*<0.001*	s(Tag)	15.5	511.0	*<0.001*
Sexmale:Tide_height intermediate	−0.523	0.051	*<0.001*				
Sexmale:Tide_height low	−1.042	0.062	*<0.001*				

**Figure 6.**
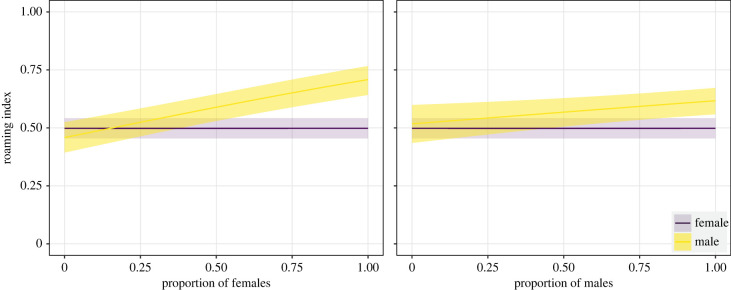
Roaming index of female and male sevengill sharks (*Notorynchus cepedianus*) as a function of the proportion of active individuals of both sexes. Solid lines represent the mean prediction and shaded areas in the 95% confidence interval.

Based on the GAMM model selection, the inclusion of the prop_f and prop_m interactions with sex and the tag as a random effect led to the best model fit for the seasonal roaming index (table 1). The final model included sex, prop_f, prop_f:sex interaction, prop_m, prop_m:sex interaction, surface temperature and tag (table 1). Only the seasonal changes in the roaming behaviour of males were affected by the proportion of active sharks of both sexes (table 2). Males showed an increased roaming index with an increasing proportion of both active females and males in the array, and this trend was more pronounced with the presence of active females (figure 6). The roaming index increased with increasing surface temperature in both sexes (electronic supplementary material, figure S3).

**Figure 7.**
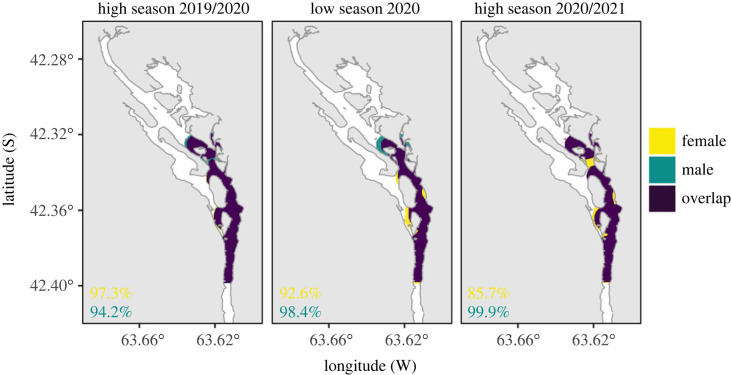
Space use overlap between female and male sevengill sharks (*Notorynchus cepedianus*). The areas correspond to the 50% contour home range. The scores of overlap (%) indicate the proportion of the area shared with the opposite sex.

The dBBMM analysis showed no differences in space use patterns between sexes nor abundance seasons, indicating a common use of CV by females and males across time (figure 7). In general, the percentage overlap of the 50% space use areas with the opposite sex was high for both females (mean = 91.9%) and males (mean = 97.5%) (figure 7).

### 3.2. Intra-day patterns

**Figure 8.**
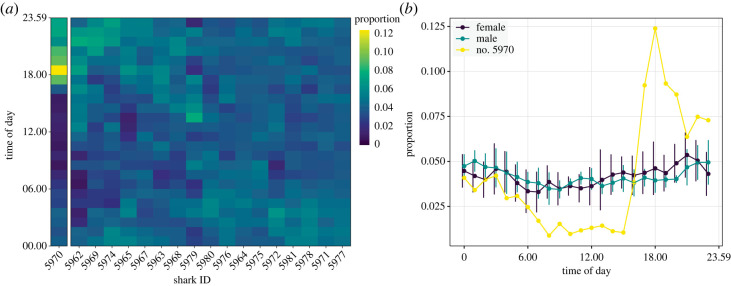
Hourly detection patterns for tagged sevengill sharks (*Notorynchus cepedianus*). The heat map shows the proportion of detections per hour of the day for each individual grouped by hierarchical clustering (*a*). The mean (dots) ± standard deviation (bars) proportion of detections for sexes and tag no. 5970 is also shown (*b*).

The circular distributions of the hourly detection patterns were non-homogeneous among individuals (Rao's test statistic = 889.4, d.f. = 53, *p*-value < 0.001), suggesting that at least one individual differed from the rest in its hourly patterns. The hourly detection frequency distributions of individuals were also non-uniform along the 24 h period (Rao's spacing test, all *p*-values < 0.001), indicating the hour of the day significantly affected detections. However, only two significantly distinct clusters were identified, one comprised one individual with a highly heterogeneous pattern of detections, and the other grouping the rest of individuals with more similar patterns (figure 8*a*). The individual with the odd pattern (no. 5970, female) was detected in greater proportions from dusk to midnight hours and in lesser proportions during daylight hours (figure 8*b*). The rest of the individuals were only detected in slightly lesser proportions during morning hours and differences in patterns between sex were not evident (figure 8*b*).

**Figure 9.**
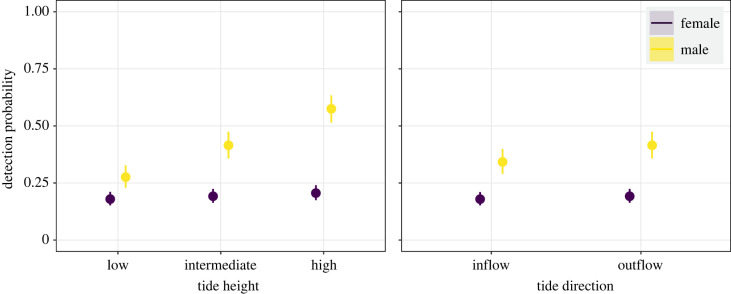
Intra-day detection probability of female and male sevengill sharks (*Notorynchus cepedianus*) as a function of tide height and direction. Points represent the mean prediction and error bars in the 95% confidence interval.

Based on the GAMM model selection, the inclusion of the tide height, tide direction and hour of the day interactions with sex, and the tag as a random effect led to the best model fit for the intra-day detection probability, although the explanatory power was low (table 1). The final model included sex, tide height, tide direction, hour of the day (and their interaction with sex) and tag (table 1). All predictors affected shark detection probability, suggesting that the tide has some effect on habitat use (table 2). Male individuals showed a higher detection probability (up to 0.5) with high tide and outflow direction, whereas females showed no changes with varying tide metrics (figure 9). The detection probability showed little increase during dawn hours for males but remained constant for females (electronic supplementary material, figure S4).

## 4. Discussion

### 4.1. Seasonal use of Caleta Valdés

A clear seasonal pattern in occurrence of *N. cepedianus* in CV was evident, with greater proportions of tagged individuals present between October and February, and lower numbers of individuals the rest of the year. This result confirmed previous coarser-scale temporal abundance patterns and site fidelity determined from fishing and BRUVs surveys [40]. During fishing surveys in the high abundance period, several sharks regurgitated chunks of elephant seals (A.M. de Wysiecki, A.J. Irigoyen, 2019 personal observation; [40]), coinciding with *N. cepedianus* seasonal use of CV largely overlapping with seasonal habitat use patterns of elephant seals. Multi-annual surveys show large aggregations of elephant seals breeding and moulting during September and mid-February [49]. The seal breeding season is characterized by a first peak in the number of adults during October and is followed by a higher peak in December corresponding to the moulting season [49]. The first peak coincided with the arrival of more sharks to CV, whereas the second peak matched the predator's highest relative abundance within the aggregation site. Since the arrival and departure of sharks and seals to the area are synchronous, the use of the area by *N. cepedianus* may be driven by the abundance of its prey. Similar patterns of predator–prey overlap in spatial use were documented in Tasmanian and suggested for the Washington coasts [23,36,39]. The peak in weanling seal pups diving for the first time in October [49] and abundance of adult seals steadily increasing a few weeks prior to peaks in shark occurrence (December) further supports seal prey as the primary driver for seasonal aggregation of *N. cepedianus* in CV [40]. This work further supports that globally, coastal areas with abundant prey are essential feeding grounds for adult *N. cepedianus* [13,33,34,39].

Sexual differences in residency patterns of *N. cepedianus* suggest more complex dynamics in the use of coastal habitats than previously thought. Although no sex differences were notable during the peak of shark occurrence, a high proportion of males departed abruptly from the receiver array by late summer. By contrast, females generally left the array more gradually towards the end of the summer, but some were detected during winter. As a consequence of losing five receivers, we could not determine whether sharks used the lower half or left CV during winter. However, because of the close distance (approx. 30 km) between lower and upper CV, the distinct drop in sharks detected on upper receivers suggests they have left for long periods (up to 183 days, mean 53 days) in winter. Similarly, sex-specific timing in use of coastal feeding grounds was evident in Tasmania, where males arrive in summer after females have already moved into coastal areas in spring [64]. Males in Tasmania also undertook northern migrations in winter, while females remained near the tagging location or moved into deeper waters [43,64]. In the north-eastern Pacific, however, the three individuals that were recorded performing long-distance movements were all females [42]. The recent recapture of a male in Rocas Coloradas (45.5°S, electronic supplementary material, figure S1) in March 2021 that was tagged approximately 490 km away in CV in November 2020 is the first direct evidence supporting large-scale movements of males to the south. These data suggest that at least males are likely to move to other semi-isolated coastal areas in the south, namely Caleta Malaspina and Ria Deseado, where *N. cepedianus* occurred in high numbers in the past between November and March [46,65]. These southern movements are expected to reverse to the north during autumn, as sharks are no longer caught in southern locations during colder months. Sharks detected on baited underwater cameras during autumn in deeper areas of the Nuevo gulf and San José gulf near CV (P. Merlo, 2020, unpublished data), further support that some individuals remain within the northern Patagonia region year-round. Similar to the year-round occurrence in coastal habitats and movements into deeper waters adjacent to coastal areas in autumn/winter documented for some female *N. cepedianus* in Tasmania [38,64]. Overall, the scale and patterns of habitat use observed in this study agree with *N. cepedianus* historical occurrence records and environmental niche analysis supporting both year-round use of coastal and shelf areas in northern Patagonia and seasonal use of coastal areas in southern Patagonia [44].

### 4.2. Movements within Caleta Valdés

Males visited more receivers in the presence of more individuals, suggesting a shift in movement behaviour during the high abundance season. The roaming behaviour of females, in contrast, was not affected by the presence of more sharks. In addition, sexes showed no clear spatial segregation in the use of CV, suggesting a common seasonal use of the area. Since elephant seals occur widespread in CV and are consumed by both sexes during the high abundance season [40], sex-based differences in roaming behaviour are unlikely to be a result of food resource partitioning. Instead, these differences could be related to different feeding strategies between sex. For example, males may traverse a larger segment of CV patroling parallel to the shoreline in search of locations in CV where elephant seals are vulnerable. Active patrol parallel to the coast to locate vulnerable seals is common in white sharks [66]. Alternatively, the increased roaming of males in the presence of females may represent mate-searching behaviour during the high abundance season. During these months, around 30% of females had fresh mating scars, indicating recent copulatory activities [40]. Females with mating scars were also recorded within bays in other *N. cepedianus* populations [38,67,68]. Whether mating occurs within CV or females with fresh mating scars move into CV after mating is still unknown. Although males surveyed in the area were actively producing sperm during spring and summer and females were simultaneously at different reproductive stages [40]. It is also possible that unreceptive females use the shallow water and the strong tidal energy in the area to avoid copulation [69]. For example, mating events in nurse sharks (*Ginglymostoma cirratum*) were recorded taking place outside the shallow study site, which suggests females may travel to deeper water to mate but stay in the shallows to avoid male harassment [70]. Given 30% of females displayed recent mating scars, CV and surrounding areas in northern Patagonia may function as mating grounds, or mating may be opportunistic while in CV to feed on abundant prey.

The absence of a diel pattern indicates that, on average, *N. cepedianus* used the area nearly evenly across the day, instead of moving in and out from the acoustic array at specific hours. Accordingly, in Norfolk Bay, Tasmania, no significant effect of time of day was found on the occurrence of *N. cepedianus* [23]. However, it remains unknown whether individuals behaved differently during the day in CV. In Norfolk Bay, *N. cepedianus* primarily appears to hunt at night [13,23]. Nocturnal movements were characterized by oscillatory swimming with faster decent rates, while diurnal movements were oriented towards substrate [23]. Behaviours suggested to be related to attacking benthic prey from above and marine mammals from below [23,71]. Given *N. cepedianus*' diverse diet [23,34–36], and the role of context in movement behaviours [41], activity patterns may vary between feeding grounds. The maximum detection probability of male *N. cepedianus* at high tide in CV suggests they are more active when water levels are higher. In CV, incoming tides passively reach elephant seals resting on the shore, and these seals show no signs of wariness (A. Irigoyen, 2019, personal observation), so sharks may be exploiting the high tide to increase access to prey. In other large coastal predators like lemon sharks (*Negaprion acutidens*), high tides allowed access to suitable foraging areas across intertidal habitats but were forced to deeper waters when tides were low [72]. Due to lost receivers, it is uncertain whether sharks used the lower half or left CV during low tides.

### 4.3. Model assumptions

One of the underlying assumptions in our inference regarding the movement behaviour of *N. cepedianus* is that mortality is negligible during the observational window. In other words, we assume that individuals that stopped being detected are purely a result of abandoning the survey area. However, individuals may also stop being detected as a result of mortality. To prevent the possible bias involved in mortality we curated the detection data in two ways: (i) we discarded the sharks that were only detected in the array within the first five days in total and (ii) we only considered detection data from the beginning of the monitoring period until each shark’s last detection (i.e. maximum residency index). During the observational window, however, sharks can be subjected to other sources of mortality after being detected for the last time. We identified three different possible sources of mortality in our study. First, tagging can increase fish mortality, which is usually known as tagging-induced mortality [73]. Second, tagged individuals may die from natural sources such as senescence, predation by killer whales [74] (e.g. killer whales enter CV and were present on a tagging day), or cannibalism, which has been reported on *N. cepedianus* [75]. Third, although *N. cepedianus* is not targeted by commercial fishing, it is caught as bycatch in many fisheries in the region (e.g. [76,77]) and thus, some levels of fishing mortality can also be expected on this species. Nevertheless, all these three sources of bias on the inference of *N. cepedianus* movements can be considered negligible. Tagging-induced mortality using internal tags in teleost fish can be high (e.g. up to approx. 40%, [78]), but it is expected to be very low in *N. cepedianus*, as individuals have shown high survival rates (approx. 97% of the tagged sharks were posteriorly detected) and minimal evidence of being tagged when recaptured [64]. Natural mortality in top predators and especially in elasmobranchs species is usually low, and particularly in the case of *N. cepedianus*, natural mortality is extremely low given captive individuals were estimated to live up to 30 years [79]. In addition, the fishing mortality of this species can be considered low given fishing is illegal in the CV area and *N. cepedianus* has little commercial value in the region. Although mortality during the observational window can be negligible, we recommend a detailed assessment of these three sources of mortality in the particular case of *N. cepedianus* and other top predators to correct any potential source of bias on the movement and behaviour inferences based on detection data.

### 4.4. Significance of Caleta Valdés

The present findings support the important role CV and potentially other key coastal habitats may play in *N. cepedianus* population dynamics and survival. The residency and movement patterns described in this study confirmed CV as a seasonal aggregation area to which adult sharks display site fidelity in the Southwest Atlantic. Similar patterns of habitat use by *N. cepedianus* of similar size were also recorded in another marine inlet 420 km to the south, Caleta Malaspina [46]. However, the marine inlet of CV is not only an important seasonal aggregation area where individuals return every year, but also an area of frequent use year-round for at least a fraction of the adult population. This suggests CV experiences favourable conditions for *N. cepedianus* and may represent an important section of its core area of distribution in northern Patagonia for both feeding and mating [44]. Other sites in the region show significant seasonal increments in *N. cepedianus* occurrence. These sites have also been suggested as essential habitat for *N. cepedianus* life stages, e.g. estuarine conditions in Partido de La Costa may facilitate feeding for young of the year [80], Bahia Anegada may function as a nursery area due to the presence of neonates, and Ria Deseado may be a secondary nursery area for larger juveniles [65]. The presence of younger life stages to the north suggests *N. cepedianus* primary nurseries necessitate warmer conditions, while coastal aggregation sites to the south provide feeding opportunities for larger individuals across colder conditions. The spatial separation between these key coastal sites (electronic supplementary material, figure S5) and the spatial continuity in historical catch records of the species [44], give support to the hypothesis that *N. cepedianus* constitutes one population in the Southwest Atlantic.

As opposed to other coastal sites in Argentina, CV is also unique because it is used only by adult individuals that likely target a specific prey, i.e. elephant seals. Notably, *N. cepedianus* is the only fish in the Southwest Atlantic preying on pinnipeds [81], and therefore likely contributing to the top-down control of their populations, an ecological role only shared with the killer whale [82]. The semi-enclosed and small area characteristics of CV, the predictable seasonal high occurrence of predator and prey, the catchability and recapture rate (7.6%) of *N. cepedianus* [40] and ability to count elephant seal numbers provides the rare opportunity to expand work in the area to focus on multi-species and broader ecosystem studies. For instance, absolute population estimates of *N. cepedianus* [22] using CV can feed into bioenergetic models to estimate parameters such as predation rates/natural mortality for fisheries and ecosystem models [33].

Information from the current study and previous work identifying essential habitats in coastal bays and inlets suggest *N. cepedianus* may be vulnerable to localized pressures. Currently, CV lays within an MPA established to preserve marine mammals and birds, while the protection of *N. cepedianus* remains circumstantial and hardly enforced. In this sense, further studies focusing on shark movements and identifying essential habitats are needed to assist the design of effective MPAs [30,83]. This, in turn, will allow for specific management to be implemented within other seasonally important areas. Overall, the life-history characteristics of *N. cepedianus*, e.g. males mature at 170 cm and females at 190 cm, reproduce every 2–3 years and give birth up to approximately 90 pups [84], and its historical decline in abundance in the Southwest Atlantic [85,86] calls for improved protection and law enforcement within key aggregation areas such as CV.

## Data Availability

All necessary data for reproducing the analyses, including detections, temperature and tidal data and relevant shape files, are accessible through the Dryad Digital Repository: https://doi.org/10.5061/dryad.fj6q5740p [87]. Data and relevant code for this research work are stored in GitHub: https://github.com/Agustindewy/Sevengill_shark_telemetry and have been archived within the Zenodo repository: https://doi.org/10.5281/zenodo.8356060 [88]. The data are provided in electronic supplementary material [89].
